# Combination Treatment of Retinoic Acid Plus Focal Adhesion Kinase Inhibitor Prevents Tumor Growth and Breast Cancer Cell Metastasis

**DOI:** 10.3390/cells11192988

**Published:** 2022-09-26

**Authors:** Ana Carla Castro-Guijarro, Fiorella Vanderhoeven, Joselina Magali Mondaca, Analía Lourdes Redondo, Felipe Carlos Martin Zoppino, Juan Manuel Fernandez-Muñoz, Angel Matias Sanchez, Marina Inés Flamini

**Affiliations:** 1 Laboratorio de Biología Tumoral, Instituto de Medicina y Biología Experimental de Cuyo (IMBECU), Consejo Nacional de Investigaciones Científicas y Técnicas (CONICET), Universidad Nacional de Cuyo, Mendoza CP 5500, Argentina; 2 Laboratorio de Transducción de Señales y Movimiento Celular, Instituto de Medicina y Biología Experimental de Cuyo (IMBECU), Consejo Nacional de Investigaciones Científicas y Técnicas (CONICET), Universidad Nacional de Cuyo, Mendoza CP 5500, Argentina; 3 Laboratorio de Oncología, Instituto de Medicina y Biología Experimental de Cuyo (IMBECU), Consejo Nacional de Investigaciones Científicas y Técnicas (CONICET), Universidad Nacional de Cuyo, Mendoza CP 5500, Argentina

**Keywords:** retinoids, FAK inhibitor, cell motility, metastasis, breast cancer

## Abstract

All-trans retinoic acid (RA), the primary metabolite of vitamin A, controls the development and homeostasis of organisms and tissues. RA and its natural and synthetic derivatives, both known as retinoids, are promising agents in treating and chemopreventing different neoplasias, including breast cancer (BC). Focal adhesion kinase (FAK) is a crucial regulator of cell migration, and its overexpression is associated with tumor metastatic behavior. Thus, pharmaceutical FAK inhibitors (FAKi) have been developed to counter its action. In this work, we hypothesize that the RA plus FAKi (RA + FAKi) approach could improve the inhibition of tumor progression. By in silico analysis and its subsequent validation by qPCR, we confirmed RARA, SRC, and PTK2 (encoding RARα, Src, and FAK, respectively) overexpression in all breast cells tested. We also showed a different pattern of genes up/down-regulated between RA-resistant and RA-sensitive BC cells. In addition, we demonstrated that both RA-resistant BC cells (MDA-MB-231 and MDA-MB-468) display the same behavior after RA treatment, modulating the expression of genes involved in Src-FAK signaling. Furthermore, we demonstrated that although RA and FAKi administered separately decrease viability, adhesion, and migration in mammary adenocarcinoma LM3 cells, their combination exerts a higher effect. Additionally, we show that both drugs individually, as well as in combination, induce the expression of apoptosis markers such as active-caspase-3 and cleaved-PARP1. We also provided evidence that RA effects are extrapolated to other cancer cells, including T-47D BC and the human cervical carcinoma HeLa cells. In an orthotopic assay of LM3 tumor growth, whereas RA and FAKi administered separately reduced tumor growth, the combined treatment induced a more potent inhibition increasing mice survival. Moreover, in an experimental metastatic assay, RA significantly reduced metastatic lung dissemination of LM3 cells. Overall, these results indicate that RA resistance could reflect deregulation of most RA-target genes, including genes encoding components of the Src-FAK pathway. Our study demonstrates that RA plays an essential role in disrupting BC tumor growth and metastatic dissemination in vitro and in vivo by controlling FAK expression and localization. RA plus FAKi exacerbate these effects, thus suggesting that the sensitivity to RA therapies could be increased with FAKi coadministration in BC tumors.

## 1. Introduction

All-trans-retinoic acid (RA) is a biologically active derivate of vitamin A with an established role in cell growth, differentiation, and apoptosis [1,2]. It exerts its effects through nuclear receptors called retinoic acid receptors (RARs: RARα, RARβ, and RARγ), which form heterodimers with retinoid-X-receptors (RXR) to control the transcription of target genes. The transcription of more than 3000 human genes is associated with RA modulation due to retinoic acid response elements (RARE) in their promoter regions [1,3]. Inactivated RAR-RXR heterodimers form complexes with corepressors to block transcription by interfering with the access to chromatin [4]. In contrast, RA binding releases corepressors and thereby coactivators take their place to allow access to chromatin [5,6]. RA can also function via the non-genomic pathway. When RA binds to a fraction of the RARs pool present in the plasma membrane, they are rapidly and efficiently activated, resulting in the induction of multiple kinases and scaffold proteins involved in several signal transductions, thus leading to numerous effects in the cell [6].

Several studies highlight the antitumor activity of RA, particularly in the modulation of cell differentiation, metastasis, apoptosis, proliferation, migration, and invasion [7]. Due to its wide range of biological effects, RA is considered a promising anticancer drug. To date, the most successful use of RA in cancer therapy has been in the treatment of acute promyelocytic leukemia (APL) and precancerous lesions, such as leukoplakia, actinic keratosis, and cervical dysplasia [8,9,10]. Unfortunately, the attempt to use RA to treat the most prevalent cancers, such as breast cancer (BC), has failed, since it demonstrated controversial results [11,12]. This urges the need for studies focused on better understanding the mechanical and biological function of RA.

Metastasis is at present the leading cause of death in BC patients, it being the most challenging threat in treating advanced cancer [13,14]. The prerequisite for cancer cells to metastasize is to acquire a highly migratory and invasive phenotype. Cell migration requires the continuous and orchestrated formation and turnover of focal adhesion sites (FAs) [15]. FAs are structures involved in the attachment of cells to the extracellular matrix (ECM) and in the recruitment of cortactin, N-WASP, and Arp2/3 complex to the reorganization of actin fibers to form specialized structures that trigger cell motility [15,16]. FAs are formed by several proteins, including focal adhesion kinase (FAK), Src, paxillin, talin, vinculin, and others [15]. FAK behaves as a kinase and adapter molecule. It is considered a central regulator of cell migration since it is uniquely positioned at the convergence point of integrins and receptor tyrosine kinase signal transduction pathways, which transmit signals from the ECM to the cell cytoskeleton [17]. FAK is overexpressed in colorectal, lung, ovarian, prostate, and breast cancer, and is associated with tumor metastatic behavior [18,19,20]. 

Due to the critical role of FAK in tumorigenesis, several pharmaceutical FAK inhibitors (FAKi) have been developed and are currently being tested in clinical trials (clinicaltrials.gov, accessed on 28 May 2021) [21]. It has been suggested that FAKi need to be combined with other agents, as today they have not shown evidence of single-agent activity [22,23,24]. At present, there are no studies that focus on the combination of RA plus FAKi in BC therapy. The combination of RA and protein kinase C inhibitor has been recently suggested as a potential therapy to treat BC. These authors demonstrated prompted results of this combination in the inhibition of BC development and further encouraged the design of novel treatments employing retinoids and protein kinase inhibitors [25]. Previous research from our laboratory has demonstrated that RA inhibits adhesion and migration in BC cells [26,27,28], cellular processes where FAK plays a key role. Based on this, we hypothesized that the combination of RA plus FAKi (RA+FAKi) improves the treatment of advanced-stage BC patients. Thus, the aim of the present study was to determine the effect of RA administrated in combination with FAKi on human BC cell adhesion and migration to further learn about signaling pathways triggered by RA, particularly in those processes that are key to the development of metastasis.

## 2. Materials and Methods 

### 2.1. Cell Culture and Treatments

The human breast carcinoma cell lines SK-BR3, T-47D, MDA-MB-231 and the human cervical carcinoma cell line HeLa were obtained from the American Type Culture Collection (ATCC, Manassas, VI, USA). The murine mammary adenocarcinoma cell line LM3 was kindly provided by the Instituto de Oncología Ángel H. Roffo (Buenos Aires, Argentina) [29]. SK-BR3 and MDA-MB-231 cells were cultured in Dulbecco’s Modified Eagle’s Medium (DMEM), high glucose, supplemented with 10% fetal bovine serum (FBS), penicillin, and streptomycin. T-47D cells were grown in RPMI 1640 supplemented with L-glutamine, 10% FBS, penicillin, and streptomycin. LM3 cells were cultured in a minimum essential medium (MEM), supplemented with 10% FBS and gentamycin. HeLa cells were cultured in DMEM medium supplemented with 10% FBS, L-glutamine, penicillin, and streptomycin. All cell lines were maintained at 37 °C in an incubator with 5% CO_2_. All-trans-retinoic acid (RA) was obtained from Sigma Chemical Co (Sigma-Aldrich, St. Louis, MO, USA). RA stock solution was dissolved in dimethyl sulfoxide (DMSO) and maintained at −20 °C, protected from light in an inert atmosphere. According to our previously published results, RA was used at a final concentration of 0.1–100 μM for 72 h [26,27]. FAK inhibitor (FAKi, sc-203950) was purchased from Santa Cruz Biotechnology and was diluted in cell culture medium to the final concentration of 1–2 µM. All experiments with retinoids were performed under reduced room light conditions.

### 2.2. Bioinformatics

#### 2.2.1. Basal Gene Expression Study in Eight Cell Lines

Two microarray gene expression datasets (GSE70884 and GSE68651) were programmatically downloaded from the publicly available Gene Expression Omnibus database (ncbi.nlm.nih.gov/geo/query/acc.cgi?acc=GSE70884 and ncbi.nlm.nih.gov/geo/query/acc.cgi?acc=GSE68651. Accessed on 28 May 2021) using the R *GEOquery* package [30]. The first dataset consists of seven BC cell lines (MCF-7, T-47D, ZR75.1, BT-474, SK-BR3, MDA-MB-468, MDA-MB-231) with three replicates each. The second dataset is composed of three replicates of the MCF-10A cell line. The Combat method from the SVA package was used to combine the datasets into a single one and perform batch effects corrections on the expression values [31,32]. For expression profiling and differential gene expression (DGE) analysis, log_2_ transformation of the expression values was applied. For boxplot construction, we used the same eight cell lines mentioned above and five genes involved in RA-antitumoral effects (RARA, RARB, RARG, SRC, PTK2). For the heatmap construction and cluster analysis, from the previously obtained expression matrix, five cell lines (MCF-10A, T-47D, SK-BR3, MDA-MB-468, MDA-MB-231) and 16 genes involved in different processes related to metastasis (CFL1, ACTR2, WASL, ACTR3, CDH1, VIM, MSN, TLN1, CTNNB1, VCL, DNM2, PXN, CTTN, MMP9, MMP2, and CDH2) were selected. A *z*-score of each gene was performed to visualize the differences in gene expression patterns between each cell line. The columns were sorted based on a hierarchical cluster with average linkage and Pearson’s correlation distance. According to Silhouette dendrograms, samples were grouped into two major clusters and five minor ones representing each cell line. A differential gene expression analysis (DGE) was performed using the R *limma* package [33], comparing SK-BR3 vs. MCF-10A cell lines and T-47D vs. MCF-10A cell lines. In all cases, log_2_ fold change values were obtained associated with the exact *p*-values.

#### 2.2.2. Gene-Expression Study in RA and Control BC Cell Lines

A gene expression dataset (GSE103426) was programmatically downloaded from the publicly available Gene Expression Omnibus database (ncbi.nlm.nih.gov/geo/query/acc.cgi?acc=GSE103426. Accessed on 28 May 2021) using the R *GEOquery* package [30]. This dataset consists of two BC cell lines (MDA -MB-231, MDA-MB-468) with three replicates each, with or without RA treatment. A differential gene expression analysis (DGE) was performed using the R *limma* package [33] comparing RA treated MDA-MB-231 vs. control MDA-MB-231 BC cell lines and the RA treated MDA-MB-468 vs. control MDA-MB-468 BC cell line. In all cases, log_2_ fold change values were obtained associated with exact *p*-values.

### 2.3. Reverse Transcription—Quantitative PCR

Total RNA was isolated from MDA-MB-231 and T-47D cells using Trizol (Invitrogen Life Technologies, Thermo Fisher Scientific, Waltham, MA, USA) following the manufacturer’s instructions. The integrity of the RNA was verified by agarose gel electrophoresis. The quantity and purity of RNA were determined by employing a NanoDrop 2000 UV/Vis spectrophotometer (Thermo Scientific, Thermo Fisher Scientific, Waltham, MA, USA). Reverse transcription was performed using a universal Oligo-dT adaptor primer and M-MLV reverse transcriptase (Moloney murine leukemia virus, Promega, Madison, WI, USA) in a thermal cycler at 37 °C for 60 min. cDNA was stored at −80 °C for further analysis. Furthermore, specific primers were designed for each gene: SRC, PTK2, VIM, CTNNB1 and CTTN. Real-time PCR was performed in triplicate for each gen, using Eva Green on Corbett Research Rotor-Gene 6000 (Qiagen, Germantown, MD, USA) with the following conditions: denaturation at 95 °C for 5 min followed by 40 cycles of denaturing and annealing. Additionally, a non-template control was included on the same run. PCR efficiencies and expression of candidates were calculated using the LinRegPCR program (version 2021.1) [34]. The relative expression of the analyzed genes was normalized to the expression of β-actin.

### 2.4. Immunoblotting

Cells were harvested in lysis buffer including 100 mM Tris-HCl (pH 6.8), 4% SDS, 20% glycerol, 1 mM Na3VO4, 1 mM NaF, and 1 mM PMSF, 1 mM PIC, 1 mM PhiC. Cell lysates were separated by SDS-PAGE and transferred into PVDF membranes. Primary antibodies used were: RARα (sc-515796), RARβ (sc-552), RARγ (sc-7387), Src (sc-5266), LDH (sc-133123), p-Paxillin (sc-365020) and Paxillin (sc-31010) from Santa Cruz Biotechnology (Santa-Cruz, CA, USA); p-FAK (BD-611807) and FAK (BD-610088) from BD Transduction Laboratories; cleaved PARP1 (ab32064) from Abcam (Cambridge, UK), active caspase-3 (bs-0081R) from Bioss. Secondary antibodies (Bioss Antibodies, Woburn, MA, USA) used were: anti-rabbit IgG-HRP (sc-2357), anti-mouse IgG-HRP (sc-358914) and anti-goat IgG-HRP (sc-2354) from Santa Cruz Biotechnology. Primary and secondary antibodies were incubated using the standard techniques. Immunodetection was accomplished using enhanced chemiluminescence and was recorded with a quantitative digital imaging system (Chemidoc XRS with Image Lab, Bio-Rad, Hercules, CA, USA).

### 2.5. Cell Adhesion Assay

Cells were exposed to RA (1–10 μM), FAKi (1 μM) or their combination RA (1 μM) plus FAKi (1 μM) for 72 h. Fifteen thousand T-47D cells/well, fifty thousand LM3 cells/well, and twenty thousand HeLa cells/well were seeded into 96-well plates previously coated with 1% sterile gelatin (Sigma-Aldrich). Cells were incubated at 37 °C for 2 h. Non-adherent T-47D cells were removed by gentle washing with PBS. The attached cells were fixed with 4% paraformaldehyde and stained with 10% ethanol/crystal violet for 20 min. Images were captured and counted in ten randomly chosen fields per well using a Nikon Eclipse E200 microscope coupled to a high-resolution CCD digital camera (Nikon, Tokyo, Japan), as previously described [26]. Cell adhesion was calculated as a percentage of attached treated cells compared to untreated cells.

### 2.6. Wound Healing Assay

A scratch wound assay was conducted to assess the influence of RA (1–10 μM), FAKi (1 μM) or their combination RA (1 μM) + FAKi (1 μM) for 72 h on cell migration. T-47D, LM3, and HeLa cells were seeded in 24-well plates and incubated until 80% confluence. Wounds were made in the monolayers by scratching the surface with a pipette tip (10 µL) as uniformly and as straight as possible. The cells were washed and the treatments were prepared. Ten μM Cytosine β-D-arabinofuranoside hydrochloride (Sigma Aldrich), an inhibitor of DNA strand separation that prevents cell division, was added to each well. Cell migration was monitored for 72 h. Digital images from cells were taken with a 16× objective. The distance of migration was analyzed by phase-contrast microscopy and closed areas were quantified using ImageJ software. Cell migration was calculated as a percentage of migrated area in treated cells compared to untreated cells.

### 2.7. Cell Viability

The MTT [3-(4,5-dimethylthiazol-2-yl)-2,5-difeniltetrazol] (Sigma-Aldrich) was dissolved in DMSO to a final concentration of 5 mg/mL. The working solution was 0.5 mg/mL MTT. LM3 and T-47D cells were seeded into 96-well plates at a density of forty-five thousand cells/well and twenty-five thousand cells/well respectively. Treatment with RA (0.01–100 μM), FAKi (0.5–2 μM), and/or the combination of both drugs was performed. After 72 h, the medium was removed and the cells were incubated with 100 μL MTT/well for 4 h. The MTT was then removed and the formazan crystal rings were dissolved in 100 μL DMSO. Absorbance at 570 nm was measured by using a microplate reader (MULTISKAN EX; Thermo Scientific). Cell viability was calculated as a percentage of viability in treated cells compared to untreated cells.

### 2.8. Analysis of Drug Interactions

CompuSyn software was used to characterize the pharmacological interaction produced by treatments with 0.01–100 μM RA plus 0.5–2 μM FAKi in LM3 and T-47D cells. This software utilizes the combination index (CI) as a method for quantitation of synergism and antagonism in drug combinations based on the mass-action law designed by Chou and Talalay [35,36]. Synergism and antagonism were defined as a more or a less than expected additive effect, respectively. CI and affected fraction (FA) levels were calculated from the effects of varying doses on cell viability inhibition rates in the MTT assay. CI = 1 denotes an additive effect, CI < 1 indicates synergism, and CI > 1 indicates antagonism. The affected fraction (FA, range 0–1) is obtained by the following equation: 1 − (% survival or unaffected fraction/100%), with 1 corresponding to 100% cell survival.

### 2.9. Cell Immunofluorescence

LM3 cells were grown on coverslips at a density of twenty thousand cells/coverslip and exposed to RA (1 μM), FAKi (1μM) or their combination RA (1 μM) + FAKi (1 μM) for 72 h. Cells were fixed with 4% paraformaldehyde for 30 min and permeabilized with 0.1% triton for 5 min. Blocking was performed with 3% bovine serum albumin for 30 min at room temperature. The cells were then incubated with an antibody against FAK (BD-610088) (BD Transduction Laboratories, Franklin Lakes, NJ, USA) overnight at 4 °C. After washing, cells were incubated with goat anti-mouse IgG-Alexa Fluor 488 (A-11001, Invitrogen) for 90 min at room temperature. The cells were then incubated with Texas Red-Phalloidin (Sigma-Aldrich) for 30 min. After washing, the nuclei were counterstained with 4′-6-diamidino-2-phenylindole (DAPI, Sigma-Aldrich), and coverslips were mounted with Vectashield mounting medium (Vector Laboratories, Burlingame, CA, USA). Immunofluorescence was visualized using a Nikon Eclipse E200 microscope and recorded with a high-resolution DP70 Olympus digital camera and examined under fluorescence microscopy (FV1000 Olympus Confocal Microscope) with a Paplon 60× lens and the FV 10-ASW 1.7 software (Olympus, Japan). To perform the quantitative analysis, we measured the mean fluorescence (total fluorescence intensity divided by the number of pixels measured, which is the parameter most similar to concentration, defining regions of interest (ROI) and performing image segmentation for protein localization quantification using ImageJ Software. Briefly, using an image of a whole-cell marker, Texas Red-Phalloidin, we generated a binary mask to define our regions of interest and measured mean fluorescence only in this section. Previously, background fluorescence was removed [37]. A minimum of 50 cells per condition were analyzed.

### 2.10. Animals

The in vivo studies were conducted with female BALB/c mice between two to four months old and 20–25 g in weight, purchased from the Instituto de Medicina y Biología Experimental de Cuyo (IMBECU, Mendoza, Argentina). They were randomly assigned into four groups and housed in boxes of five animals each in a climate-controlled room, with free access to food and water and a 12 h light/12 h darkness schedule. All animals were cared for following the Guiding Principles in the Care and Use of Animal of the U.S. National Institute of Health. The Institutional Animal Care and Use Committee of the School of Medical Science, Universidad Nacional de Cuyo (UNCuyo), Mendoza, Argentina (Protocol approval N°63/2015) approved all procedures.

Groups were as follows (*n* = 5 for group, 20 animals in each independent experiment)

(1) CONTROL (indicated as CON): animals inoculated with LM3 cells received an empty subcutaneous silastic pellet.
(2) RA: animals inoculated with LM3 cells received a subcutaneous silastic pellet containing RA (10 mg).
(3) FAKi: animals inoculated with LM3 cells (pretreated with FAKi) and received an empty subcutaneous silastic pellet.
(4) RA plus FAKi (indicated as RA+FAKi): animals inoculated with LM3 cells (pretreated with FAKi) and received a subcutaneous silastic pellet containing RA (10 mg).

Animals were euthanized by cervical dislocation at the experimental endpoint or when they showed signs of suffering (human endpoint): loss of >20% of the initial weight, ulcers, or when one of the tumor diameters exceeded 10 mm. Whenever a cellular injection was performed, cell viability was higher than 95% as determined by the trypan blue exclusion test. The order of injection of different groups was randomized to eliminate any difference that may bias the outcome.

#### 2.10.1. Orthotopic Tumor Growth

Mice were injected subcutaneously into the fourth mammary gland with three hundred thousand LM3 cells, treated or not with FAKi (1 μM for 72 h). Immediately, animals of groups 1 and 3 received an empty subcutaneous silastic pellet and the animals of groups 2 and 4 received a subcutaneous silastic pellet containing RA (10 mg). Mice were monitored daily and tumor diameters were measured twice weekly with a sliding caliper. Tumor volume was calculated with the formula D × d2/2 (where d is the smallest diameter and D is the largest) for assessment of growth rate. Survival was monitored up to 45 days. Mice were sacrificed at the experimental endpoint (45 days post-inoculation) or when animals showed signs of suffering, such as ulcers, tumor volume greater than 10 mm and/or severe weight loss. Kaplan–Meier and log-rank tests were carried out to determine differences in survival times between experimental groups. Animals were necropsied to extract the tumors, which were fixed and embedded in paraffin for subsequent histopathological studies. These in vivo tests were performed in duplicate (*n* = 5 per group), with 20 animals in each independent experiment.

#### 2.10.2. Experimental Lung Metastasis Assay

The animals of groups 1 and 3 received an empty subcutaneous silastic pellet and the animals of groups 2 and 4 received a subcutaneous silastic pellet containing RA (10 mg). After a week, mice were inoculated in the tail vein with three hundred thousand LM3 cells, pretreated or not with FAKi (1 μM for 72 h). Twenty-one days post-inoculation of tumor cells, mice were euthanized and necropsied. The lungs were ablated and fixed in Bouin’s solution to record the number of superficial lung nodules in each case under a dissecting microscope. These in vivo tests were performed in duplicate *n* = 5 per group, with 20 animals in each independent experiment. 

### 2.11. Histological Analysis

For immunohistochemistry, orthotopic tumors were fixed in 4% formalin, embedded in paraffin and 4 µm sections were mounted on glass slides. Antigen unmasking was carried out in a 0.01 M citrate buffer (pH 6.0) at 100 °C for 30 min. Endogenous peroxidase activity was reduced by 30 min incubation with hydrogen peroxide (0.01%). The blockade was carried out with 10% skim milk for 1 h. The primary antibody Ki-67 (15,580, Abcam) was incubated overnight at 4 °C. As a detection method, we used the biotin-streptavidin-peroxidase system. Diaminobenzidine was used as a chromogen substrate. Slides were lightly counterstained with hematoxylin and observed with an Eclipse E400 microscope (Nikon). We analyzed five randomly selected samples from each group. Five fields were randomly considered for scoring. A minimum of 500 cells per tissue were counted.

### 2.12. Statistical Analysis

Statistical analysis of the data was performed with one-way analysis of variance (ANOVA) followed by Tukey-Kramer Multiple-Comparisons or Kruskal-Wallis test using GraphPad Prism 5.03 software (CA, USA). The Kaplan-Meier and log-rank tests were used to estimate survival time after treatments and to compare experimental groups. *p* < 0.05 was considered statistically significant. All values were expressed as mean ± standard error (SD) of three independent experiments. All bioinformatics analyses were performed using R version 4.0.4 in a Windows environment with Intel Core i7 with 32 GB of RAM (AZ, USA). 

## 3. Results

### 3.1. Differential Gene Expression Analysis of Cancer-Related Genes among BC Cells

**Figure 1:**
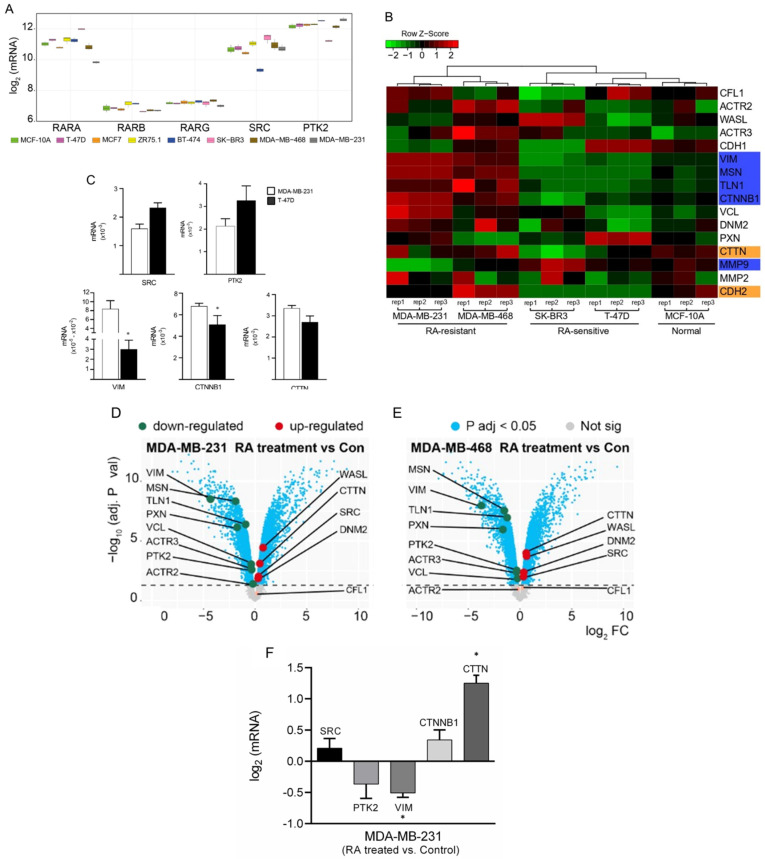
Expression of genes involved in metastasis process in a panel of BC cells. (**A**) Boxplot showing the gene expression patterns of five genes of interest: RARA, RARB, RARG, SRC, and PTK2 (encoding RARα, RARβ, RARγ, Src, and FAK respectively). Each color represents a breast cell line (MCF-10A, MCF-7, T-47D, ZR75.1, BT-474, SK-BR3, MDA-MB-468, MDA-MB-231). (**B**) Gene expression heatmap of 16 genes involved in different processes related to metastasis. The MCF-10A, T-47D, SK-BR3, MDA-MB-468, and MDA-MB-231 cell lines were used. Expression patterns of genes are represented in a green-red scale where low expression levels are in green and high expression levels in red. By a hierarchical clustering algorithm, samples were grouped into two major clusters and five minor ones that represent each cell line. (**C**) SRC, PTK2, VIM, CTNNB1 and CTTN expression in T-47D and MDA-MB-231 cells was validated with qPCR. Normalization was done with respect of the expression of β-actin. qPCR was performed in triplicate for each gene. * = *p* < 0.05 vs. MDA-MB-231 cells (**D**,**E**) Volcano plots of differential gene expression analysis achieved by limma R method. In the *x*-axis the log_2_ fold change (FC) of RA-treated MDA-MB-231 vs. control MDA-MB-231 BC cell; and RA-treated MDA-MB-468 vs. control MDA-MB-468 BC cell. Genes with adjusted *p*-value < 0.05 are indicated above the horizontal dashed line. Upregulated genes are shown in red and down-regulated genes are shown in green. (**F**) SRC, CTNNB1, CTTN, PTK2 and VIM expression was normalized with β-actin. In the *x*-axis the log2 fold change of RA-treated MDA-MB-231 vs. control MDA-MB-231. qPCR was performed in triplicate for each gene.

Published results of different trials show that the use of retinoids in BC patients has controversial results. Taking into account that RA exerts its effects via RARs and that Src-FAK are critical proteins involved in cancer progression, we initially explored the expression profiles of RARA, RARB, RARG, SRC, and PTK2 in different types of human BC cells (T-47D, MCF7, ZR75.1, BT-474, SK-BR3, MDA-MB-468, and MDA-MB-231) and a normal breast cell line (MCF-10A). For this purpose, we performed a transcriptome analysis using two datasets from the publicly available Gene Expression Omnibus database (GSE70884 and GSE68651). We found that RARA (RARα), SRC (Src), and PTK2 (FAK) are highly expressed in all breast cells. MDA-MB-231 and SK-BR3 have the lowest and highest level of RARA expression, respectively. RARB and RARG are expressed at similarly low levels in all breast cell lines (Figure 1A).

We next compared the expression of sixteen genes between RA-resistant (MDA-MB-231 and MDA-MB-468), RA-sensitive (SK-BR3 and T-47D) BC cells, and a normal breast cell line (MCF-10A). This set of genes was selected since they are biomarkers of different processes involved in BC metastasis such as MSN (moesin), CFL1 (cofilin) (*actin cytoskeleton remodeling/depolymerization process*); VCL (vinculin), TLN1 (talin), PXN (paxillin), DNM2 (dynamin) (*FAs dynamics*); CTTN (cortactin), WASL (family of the Wiskott Aldrich Syndrome Proteins, WASP), ACTR2 (Arp2 subunit), ACTR3 (Arp3 subunit) (*actin nucleation*); CDH1 (cadherin-1), VIM (vimentin), CDH2 (cadherin-2), CTNNB1 (β-catenin) (*Epithelial-Mesenchymal Transition, EMT*); and MMP2, MMP9 (matrix metalloproteinases 2 and 9) (*invasion/extracellular matrix disassembly*). All samples were sorted based on a hierarchical cluster algorithm with average linkage and Pearson’s correlation distance. According to Silhouette dendrograms, analysis samples were grouped into two major clusters. In one branch, it groups MDA-MB-231 and MDA-MB-468 (RA-resistant) cell lines; in the other, it groups SK-BR3, T-47D (RA-sensitive), and MCF-10A (normal) cell lines. Figure 1B shows genetic similarities between RA-resistant BC cell lines and, on the other hand, between RA-sensitive BC cell lines and the normal ones. In general, the selected genes show low expression in normal and RA-sensitive cells compared to the same genes that are overexpressed in RA-resistant cells. Furthermore, we observed that the expression of VIM, MSN, TLN1, and CTNNB1 is markedly different between RA-sensitive and RA-resistant BC cells. In RA-resistant cells, these genes are overexpressed; meanwhile, in RA-sensitive cells, they are underexpressed. We noted that the expression of these genes is similar between RA-sensitive cells and the normal breast cell line MCF-10A (Figure 1B, *genes marked in blue*). MMP9 expression is low in RA-resistant compared with RA-sensitive and normal lines (Figure 1B). Additionally, we found that the expression of CTTN and CDH2 is overexpressed in RA-resistant and normal breast cells compared to RA-sensitive (Figure 1B, *genes marked in orange*). Altogether, this suggests that VIM, MSN, TLN1, CTNNB1, CTTN, MMP9, and CDH2 are promising candidates to be studied as markers of RA response. Bioinformatics data were validated measuring SRC, PTK2, VIM, CTNNB1 and CTTN by qPCR in control T-47D (RA-sensitive) and MDA-MB-231 (RA-resistant) BC cells (Figure 1C).

To further elucidate whether genes are associated with RA-resistance, we performed a differential gene expression analysis in control and RA-treated MDA-MB-468 and MDA-MB-231 BC cells. In Figure 1D,E, both cells show the same behavior. RA administration (100 nM, 18 h) modulated the expression of all the genes analyzed, except ACTR2 and CFL1. Genes like WASL, CTTN, SRC, and DNM2 were upregulated (Figure 1D,E, *genes marked in red*), while VIM, MSN, TLN1, PXN, VCL, ACTR3, and PTK2 were downregulated (Figure 1D,E, *genes marked in green*). We confirmed by qPCR that in MDA-MB-231 cells the administration of 100 nm RA during 18 h upregulated the expression of SRC, CTNNB1 and CTTN while PTK2 and VIM were downregulated (Figure 1F).

These results suggest that RA-resistance would reflect the deregulation of most RA-target genes, mainly those encoding components from the signaling of the Src-FAK pathway. 

We also examined the differential gene expression between SK-BR3/T-47D and normal mammary cell line MCF-10A. We used a gene expression dataset from the publicly available Gene Expression Omnibus database (GSE103426). The volcano diagrams show that both BC cells exhibit a decreased expression of VIM, CDH2, and MSN compared to MCF-10A (Supplementary Figure S1, *genes marked in green*). We also found that SK-BR3 has an increased expression of RARA, WASL, SRC, and MMP9 compared to MCF-10A (Supplementary Figure S1A, *genes marked in red*), while T-47D has an increased expression of CDH1 and PXN (Supplementary Figure S1B, *genes marked in red*).

### 3.2. RA Reduces Cell Adhesion and Migration in Tumoral Cells

**Figure 2:**
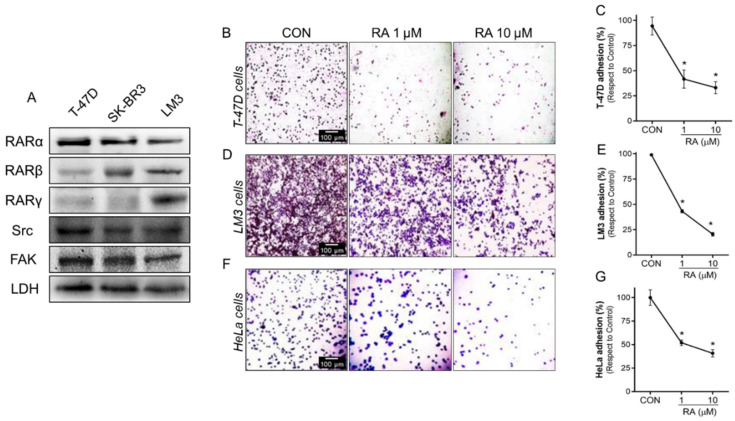

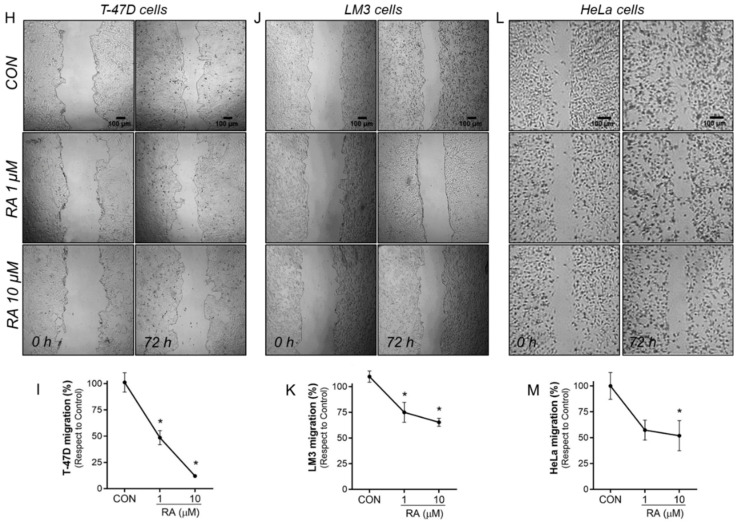
RA reduces cancer cell adhesion and migration. (**A**) Whole-cell lysates from human BC cell lines T-47D, SK-BR3, and a murine BC cell line LM3 were evaluated to determine the expression of RARα, RARβ, RARγ, Src and FAK by western blot assay. LDH expression was used as a protein loading control. A representative blot is shown. (**B**,**C**) T-47D, (**D**,**E**) LM3, and (**F**,**G**) HeLa cells were treated with RA (1–10 μM) treatment for 72 h. After the treatment, cells were seeded into 96-well plates previously covered with gelatin and a cell adhesion assay was performed. Representative images and percentages of adhered cells are shown. (**H**,**I**) T-47D, (**J**,**K**) LM3 and (**L**,**M**) HeLa cells were exposed to RA (1–10 μM) for 72 h and cell migration by wound-healing assay was performed. Representative images are shown. Gap closure was quantified by the use of the ImageJ software. Adhesion and migration values are presented as a percentage of control. * = *p* < 0.05 vs. control, CON. All experiments were performed in triplicates with consistent results.

To confirm the role of RA in inhibiting tumor progression, we first verified the expression of RARs, Src, and FAK in T-47D, SK-BR3, and LM3 by western blot analysis. We found that RARα, RARβ, RARγ, Src, and FAK were present in all BC cell lines analyzed (Figure 2A and Supplementary Figure S2).

Then, we evaluated the RA effect on cell adhesion and migration in human T-47D and murine LM3 BC cell lines. In both models, treatment with RA (1–10 μM) for 72 h markedly decreased cell adhesion to an extracellular matrix-like surface by 58–79%, respectively, compared to the control group (Figure 2B–E). Likewise, the administration of RA (1–10 μM) during 72 h reduced cell migration by 52–88% in T-47D and by 25–35% in LM3 cells (Figure 2H–K). Furthermore, we noted that RA inhibitory effects are extrapolated to other cancer cells. Treatment with RA (1–10 μM) for 72 h in the human cervical carcinoma cell line HeLa significantly inhibited cell adhesion (Figure 2F,G) and migration (Figure 2L,M) in a dose-dependent manner. 

### 3.3. Treatment with RA Plus FAKi Reduces RARα, FAK, and Paxillin Expression Decreasing LM3 Cells Viability via Caspasa-3 and PARP1 Cleavage

**Figure 3:**
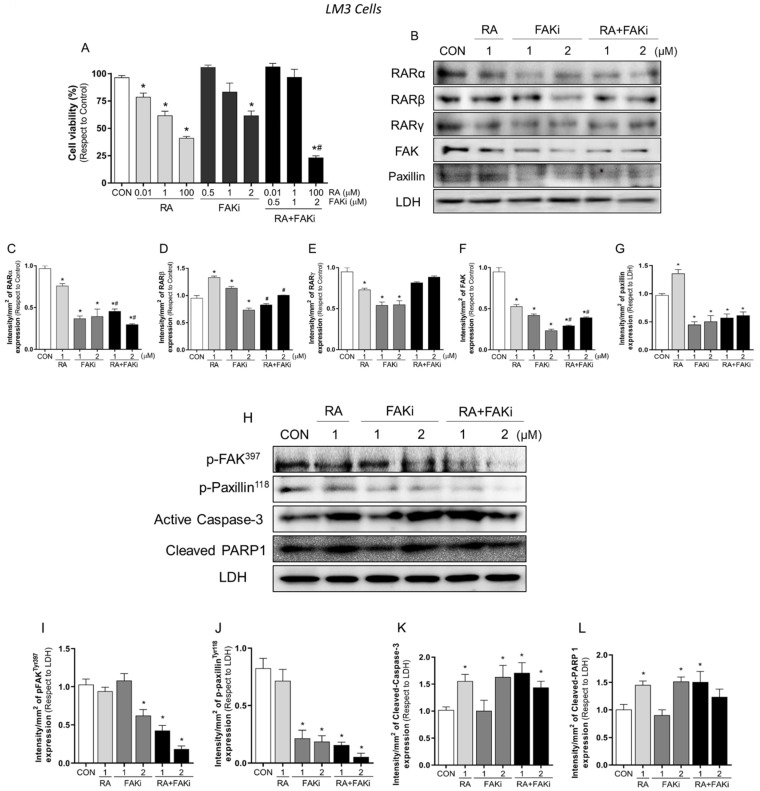

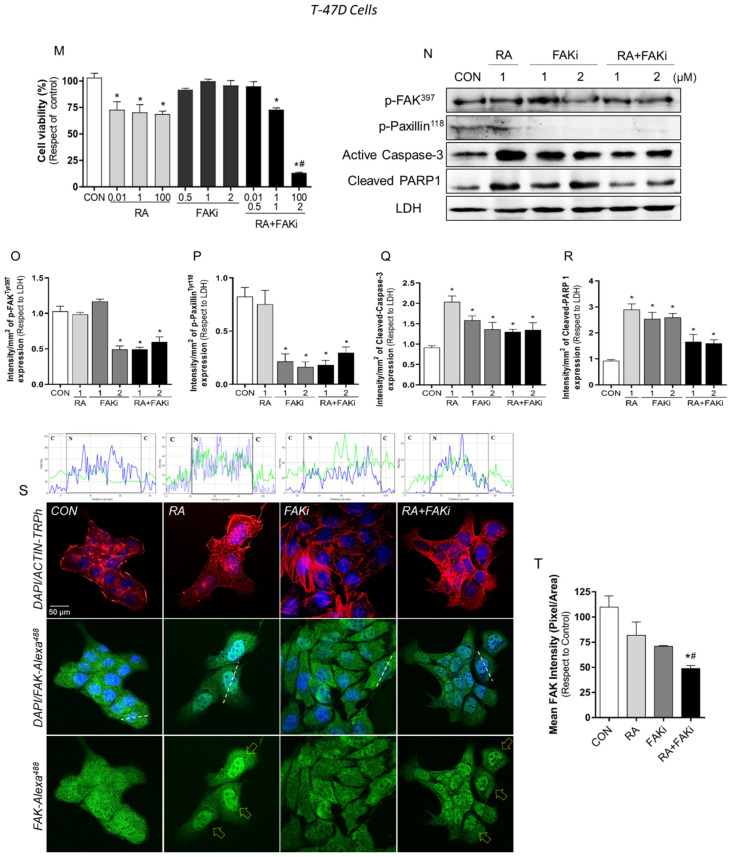
LM3 and T-47D cells viability and apoptosis. (**A**) LM3 cells were treated with increasing doses of RA (0.01–100 μM), FAKi (0.5–2 µM), and their combinations (0.01 μM RA + 0.5 µM FAKi), (1 μM RA + 1 µM FAKi) and (100 μM RA + 2 µM FAKi) for 72 h. The results were expressed as a percentage (%) of surviving cells compared to the control (CON, 100% survival). (**B**) LM3 cells were exposed with RA (1 μM), FAKi (1–2 µM) and their combinations (1 μM RA + 1 µM FAKi), (1 μM RA + 2 µM FAKi) for 72 h, and expression of RARα, RARβ, RARγ, FAK and Paxillin were analyzed by western blot assay. LDH expression is shown as a loading control. (**C**–**G**) Densitometric quantifications of RARα, RARβ, RARγ, FAK and Paxillin bands. Intensity values were adjusted to the corresponding intensity values of LDH and then normalized to the control. (**H**) LM3 cells were exposed with RA (1 μM), FAKi (1–2 µM) and their combinations (1 μM RA + 1 µM FAKi), (1 μM RA + 2 µM FAKi) for 72 h and expression of p-FAK^Tyr397^, p-Paxillin^Tyr118^, active caspase-3 and cleaved PARP1 were analyzed by western blot assay. LDH expression is shown as a loading control. (**I**–**L**) Densitometric quantifications of p-FAK^Tyr397^, p-Paxillin^Tyr118^, active caspase-3 and cleaved PARP1 bands. Intensity values were adjusted to the corresponding intensity values of LDH and then normalized to the control. (**M**) T-47D cells were treated with increasing doses of RA (0.01–100 μM), FAKi (0.5–2 µM), and their combinations (0.01 μM RA + 0.5 µM FAKi), (1 μM RA + 1 µM FAKi) and (100 μM RA + 2 µM FAKi) for 72 h. The results were expressed as a percentage (%) of surviving cells compared to the control (CON, 100% survival). (**N**) T-47D BC cells were exposed with RA (1 μM), FAKi (1–2 µM) and their combinations (1 μM RA + 1 µM FAKi), (1 μM RA + 2 µM FAKi) for 72 h and expression of p-FAK^Tyr397^, p-Paxillin^Tyr118^, active caspase-3 and cleaved PARP1 were analyzed by western blot assay. LDH expression is shown as a loading control. (**O**–**R**) Densitometric quantifications of p-FAK^Tyr397^, p-Paxillin^Tyr118^, active caspase-3 and cleaved PARP1 bands. Intensity values were adjusted to the corresponding intensity values of LDH and then normalized to the control. (**S**) LM3 cells were exposed to RA (1 μM), FAKi (1µM) or their combinations (1 μM RA + 1 µM FAKi) for 72 h. An immunofluorescence assay was performed, murine cells were stained with anti-FAK linked to Alexa Fluor 488 (green), filamentous actin was stained with phalloidin linked to Texas Red (red), and nuclei were counterstained with DAPI (blue). The images were examined under fluorescence microscopy (FV1000 Olympus Confocal Microscope). The dotted white line on the cells indicates the area analyzed in the graphs. The y-axis displays the intensity of fluorescence and the x-axis shows the pixels. C (cytoplasmic) and N (nuclear) areas indicate the parts of the graph corresponding to the cytoplasmic and nuclear areas. Fluorescent intensity levels are represented in blue (DAPI, nucleus) and green (FAK- Alexa488). Yellow arrows indicate nuclear FAK re-localization. (**T**) Quantification of mean FAK intensity in pixels per area in the different conditions. Results are expressed as a percentage of mean FAK intensity ± SD compared to the control cells. * = *p* < 0.05 vs. control, CON. # = *p* < 0.05 vs. RA. Representative images are shown. All experiments were performed in triplicate with consistent results; representative images are shown.

Due to the fundamental role of RA and FAK in tumor progression, we next evaluated whether the combined treatment of RA with the specific inhibitor of FAK (FAKi) improves the inhibition of tumorigenesis. First, we tested the sensitivity of LM3 and T-47D to drugs. We performed an MTT assay using a dose range of RA (0.01–100 μM) and FAKi (0.5–2 µM) and their combinations for 72 h. We observed that all doses of RA treatment decreased cell viability in both cell lines, meanwhile treatment with FAKi only decreased 38% of cell viability at the highest concentration (2 μM) in LM3 cells. We noted that LM3 cells are more sensitive to RA and FAKi than T-47D cells. A synergistic effect was observed in the inhibition of cell viability when combined treatments were administered at the highest concentration in LM3 and T-47D cells (Figure 3A,M). Compusyn software was then used to examine the degree of drug interaction when treating cells with both drugs simultaneously. The results indicated that RA+FAKi exerted a synergistic effect when 77% and 87% of cell growth inhibition was reached at the highest concentration tested in LM3 and T-47D cells, respectively, associated with a CI inferior to 1, as shown in Supplementary Figure S3. This implies that the interaction between the two drugs produced a more significant effect than expected for the sum of the treatments.

Next, to further study the anti-proliferative effects of RA and FAKi; LM3 and T-47D cells were treated for 72 h with RA (1 μM), FAKi (1–2 μM), and their combinations and the expression of RARs, FAK, paxillin and the apoptotic markers active caspase-3 and cleaved PARP1 were analyzed. In LM3 cells, we found that all treatments induced the downregulation of RARα, FAK, and paxillin compared to the control. The RA + FAKi combinations resulted in higher downregulation than RA alone (Figure 3B,C,F,G). On the other hand, we observed that the administration of RA induced the upregulation of RARβ, while the treatment with FAKi (2 μM) decreased its expression compared to the control group. Moreover, RA + FAKi reduced RARβ expression compared to RA but did not affect its expression compared to the control (Figure 3B,D). Additionally, RA and FAKi reduced RARγ expression as a single agent; however, their combinations did not affect it (Figure 3B,E).

Regarding apoptosis, we observed that in LM3 and T-47D cells, RA, FAKi (2µM) and the combined treatments induced an increase in apoptosis markers of active caspase-3 and cleaved PARP1 compared to control cells; the combined treatment did not enhance this effect (Figure 3H,K,L,N,Q,R).

Since the action of FAK inhibitors blocks the specific autophosphorylation and activation of FAK kinase, we investigated whether inhibition with FAKi blocks the downstream pathway of FAK. We therefore analyzed the phosphorylation of FAK and its target protein, paxillin. We confirmed that in LM3 and T-47D cells, FAKi (2 μM) and both RA + FAKi combined treatments reduced FAK and paxillin’s phosphorylation and consequent activation (Figure 3H–J,N–P). This suggest that the inhibition of FAK and paxillin persist during long periods of time.

We also performed an immunofluorescence assay on LM3 cells treated with RA (1 μM), FAKi (1 μM), and their combination for 72 h to reveal the expression and subcellular localization of FAK. We visualized that FAK is homogeneously and diffusely localized throughout the cytoplasm in control cells, whereas treatment with RA and RA + FAKi triggers FAK nuclear relocalization (Figure 3S and Figure S4). This was associated with a significant increase of nuclear FAK intensity, quantified by analyzing the pixel intensity in a line including the cell cytoplasmic and nuclear areas (Figure 3S, *boxes*). Furthermore, we observed in all treatments the longitudinal arrangement of actin fibers, typical characteristics of a static cell phenotype, suggesting that drug administration affects the reorganization of the actin cytoskeleton (Figure 3S and Figure S4), preventing the cell from being able to drive its motor machinery impairing BC cell motility. In parallel, we also confirmed that the combined treatment significantly reduced FAK expression (mean intensity, pixel/area) (Figure 3T).

### 3.4. RA and FAKi Combination Improve LM3 Adhesion and Migration Inhibition 

**Figure 4:**
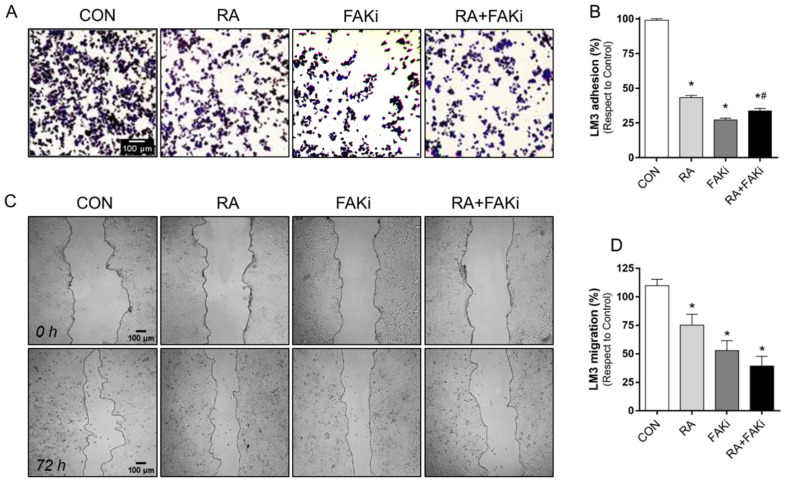
RA plus FAKi controls LM3 cell adhesion and migration. LM3 cells were treated with 1 μM RA, 1 µM FAKi or their combinations 1 μM RA + 1 µM FAKi for 72 h and cell adhesion and migration assay was performed. (**A**,**B**) Representative images and percentages of LM3 cells adhered to gelatin and (**C**,**D**) LM3 cell migration are shown. The gap closure was quantified by the use of ImageJ software. Adhesion/migration results were expressed as a percentage of attached/migrated cells vs. control cells, CON. * = *p* < 0.05 vs. control, CON. # = *p* < 0.05 vs. RA. Measures are from three independent experiments.

We tested the ability of LM3 cells to adhere to an ECM substrate after RA (1 μM), FAKi (1 μM), and their combination over 72 h. We showed that treatment with RA, FAKi, and RA + FAKi reduced cell adhesion compared to the control. The RA plus FAKi produced a more significant inhibition than RA alone (Figure 4A,B). In parallel, we exposed cells to RA, FAKi, and RA + FAKi and we monitored cell motility for 72 h. We found a significant inhibition of cell migration in all conditions tested compared to the control, reaching the major inhibition of migration in the combined treatment (Figure 4C,D).

### 3.5. RA Plus FAKi Reduces Tumor Growth and Metastasis, Increasing Mice Survival

**Figure 5:**
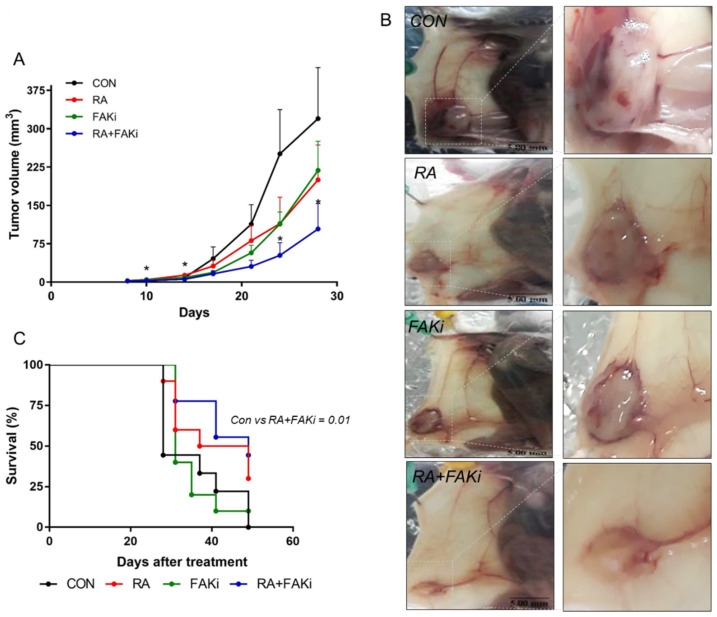

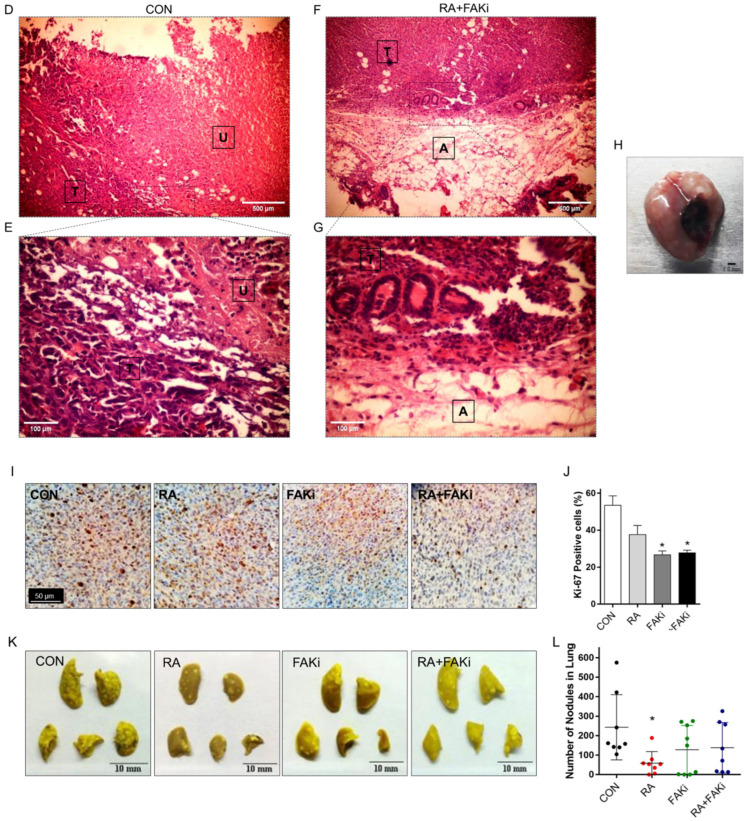
Combination of RA plus FAKi in a murine experimental model. An orthotopic tumor growth assay was performed inoculating LM3 cells pretreated or not with FAKi (1 µM, 72 h) into BALB/c mice. Animals also received a subcutaneous silastic pellet containing RA (10 mg) or an empty pellet. (**A**) Tumor diameters were measured twice a week and used to calculate tumor volume. Each data point represents the mean ± SD, * = *p* < 0.05 vs. control, CON. (**B**) Representative images are shown. A dotted square indicates tumors. (**C**) Kaplan-Meier analysis of animal endpoint survival. Each color represents a different experimental group: control group (black), RA group (red), FAKi group (green), and RA + FAKi group (blue). (**D**–**H**) Effect of RA plus FAKi on histopathological features of LM3 orthotopic tumors. Tumors were fixed in 10% formalin and slides were stained with hematoxylin and eosin (T: Tumoral tissue, U: Ulcerated tissue, and A: Adipose tissue). (**D**,**F**) are images acquired at 100x magnification. (**E,G**) are the same sections with a 400× magnification. (**H**) Representative image of an ulcerated tumor. (**I**,**J**) Images and the percentage of positive Ki-67-mammary tumor of LM3 cells compared to the control, CON; for each experimental condition are shown. * = *p* < 0.05 vs. control, CON. (**K**,**L**) An experimental metastasis assay was performed by inoculating LM3 cells, pretreated or not with FAKi (1µM, 72 h), in the lateral tail vein of mice carrying a subcutaneous silastic pellet containing RA (10 mg) or an empty pellet. Twenty-one days post-inoculation, mice were sacrificed and the number of lung nodules was recorded. Each data point represents the number of lung nodules per animal. Each color denotes a different experimental group: control group (black), RA group (red), FAKi group (green), and RA + FAKi group (blue). Representative images are shown. The figure shows the results of one experiment representative of two independent assays. * = *p* < 0.05 vs. control, CON.

Finally, we performed a murine in vivo model to determine correlation with the in vitro results. Tumor growth was evaluated by the orthotopic tumor growth assay consisting of inoculating murine LM3 cells, treated or not with FAKi (1 μM, 72 h), in the mammary gland BALB/c mice bearing RA or not bearing it (10 mg)-subcutaneous pellet. We observed reduced tumor growth in animals that received RA systemically through a slow-release pellet (RA group), and animals where FAK was pharmacologically inhibited (FAKi group); however, in the combined treatment, RA + FAKi induced a more potent inhibition in tumor volume (Figure 5A,B). In accordance with this, RA and FAKi treatment increased mice survival, but only the combination RA + FAKi was statistically significant (Figure 5C). In addition, we observed that in controls and in the FAKi group the tumors were histopathologically aggressive, even ulcerating the dermis. In these groups the tumor diameters exceeded 10 mm before 45 days of the experimental endpoint and therefore the animals had to be sacrificed because of the human endpoint. The treatment with RA and the combination RA + FAKi prevented the development of these highly proliferative tumors. Representative images of ulcers found in control animals (Figure 5D,E,H) and tumors of combined treatments are shown (Figure 5F,G). The total body weight of mice was not different in the experimental groups compared to control (Supplementary Figure S5).

On the other hand, since Ki-67 is widely used in routine pathology as an established biomarker of cell division, to assess the proliferation rate of human BC tumors, we measured Ki-67 expression by immunohistochemistry in mice tumors. We found that the percentage of positive cells for Ki-67 was markedly lower in the treatments with FAKi and RA + FAKi compared to the control group (Figure 5I,J).

Finally, we performed an experimental metastatic assay to analyze the effect of the RA + FAKi combination on the spread of tumor cells. LM3 cells, pretreated or not with FAKi (1 µM, 72 h), were injected into the tail vein of mice bearing RA or not (10 mg) silastic pellet. We found that RA significantly reduced metastatic lung dissemination. FAKi and the RA + FAKi combination presented a lower but non-significant number of lung nodules than the control group (Figure 5K,L).

## 4. Discussion

Although RA has demonstrated a potent anticancer activity, its use in solid tumors is limited by its controversial behavior, emphasizing the need for a better understanding of the RA molecular mechanism. Therefore, and taking into account that FAK is a pivotal kinase and scaffold protein that modulates cell adhesion and migration, processes involved in metastasis development, we evaluated whether a therapeutic approach based on RA administration combined with FAKi improves tumor progression inhibition.

A bioinformatics analysis and its subsequent validation by qPCR confirmed that the expression of SRC and PTK2 is high in BC cells. The overexpression of Src and FAK in many cancers is well established, and they are correlated with a poor prognosis for patients [19,38,39,40]. We also observed that RARA, encoding RARα, the central receptor that mediates the antitumor activity of RA, was expressed at different levels within BC cells. This fact coincides with RA-sensitivity reported for BC cell lines, where SK-BR3 is considered RA-sensitive and MDA-MB-231 RA-resistant based on its high and low expression of RARA, respectively [41,42,43]. We also showed that RARB and RARG, encoding RARβ and RARγ respectively, were uniformly and under-expressed in all BC. Similar results were obtained by Lu et al. (2005) [42]. This fact is not surprising since RARβ has been reported to act as a tumor suppressor [44,45]. RARβ is necessary to inhibit tumor proliferation and migration. The diminished RARB mRNA due to epigenetic alterations in BC has been widely demonstrated. It has been proposed as an epigenetic marker for BC diagnosis [26,46,47]. The role of RARγ has not been extensively studied in the context of BC. 

In addition, we showed that VIM, MSN, TLN1, CTNNB1, CTTN, MMP9, and CDH2 (encoding vimentin, moesin, talin, β-catenin, cortactin, metalloprotease-9, and N-cadherin, respectively) are differentially expressed between RA-resistant and RA-sensitive BC cells. The functional products of these genes are directly or indirectly modulated by the Src-FAK complex through interactions to trigger the metastatic process [24,48]. 

In silico analysis and qPCR results in RA-resistant MDA-MB-231 and MDA-MB-468 BC cells showed that RA treatment regulated all genes tested despite being resistant to RA. Interestingly, RA downregulated VIM, MSN, and TLN1 genes previously demonstrated to be overexpressed in RA-resistant compared to RA-sensitive cells. In addition to these genes, RA reduced the expression of other genes involved in the adhesion/migration process, such as PXN, VCL, ACTR3, and PTK2. Despite lacking RARA receptors, and therefore being resistant to RA and insensitive to the RA-induced decrease in proliferation, RA could inhibit the adhesion, migration, invasion, and, consequently, metastasis by reducing the expression of target genes involved in these events. Coyle et al. (2017) provided evidence that RA, in addition to the widely studied canonical mechanism, governs the expression of a vast number of genes in a RARE-independent way through RA-inducible transcription factors [49]. The finding marker genes of RA response based on its differential expression in RA-sensitive vs. RA-resistant BC cells is promising not only in RA-sensitive cells but also in the treatment of triple-negative BC that currently lacks a specific therapy; however, more studies on this topic are needed to reach critical conclusions, and these results are beyond the scope of the manuscript.

Collectively, these data strongly support the idea that the combination of RA plus FAKi could improve the RA therapy in treating advanced-stage BC patients. Thus, we next examined in vitro and in vivo the ability of RA + FAKi to inhibit BC progression. Numerous studies have found that RA reduces cell adhesion and migration in BC [26,27,50] and other types of cancer [51,52,53]. Our results confirm that RA inhibits cell adhesion and migration in T-47D, LM3, and HeLa cells. Disruption of cell adhesion and motility has also been reported after FAKi treatment in BC cells [54,55,56,57]. Our findings reveal that although RA and FAKi administered separately decrease adhesion and migration in LM3 BC cells, the novelty of our work is that their combination exerts a higher effect. 

We also examined the ability of RA + FAKi on in vitro LM3 and T-47D cell viability and apoptosis. We noted a synergistic effect when RA (100 μM) and FAKi (2 μM) were combined. Although no studies evaluated this combination, several reports demonstrated that RA and FAKi administrated separately inhibit BC cells’ viability [58,59,60,61]. 

The anticancer effects of RA are mainly the result of growth inhibition with the subsequent induction of apoptosis. Several studies reported the induction of apoptosis after RA treatment [25,62,63]. Hong TK & Lee-Kim YC showed that RA (1 µM, 4 days) induced apoptosis increasing caspase activity in MCF7 cells. They did not observe effects in RA-resistant MDA-MB-231 BC cells [64]. Similarly, in SK-BR3 BC cells, RA (1 µM, 2–4 days) induces caspase-3 cleavage in a time- and dose-dependent manner [65]. Additionally, a vast bibliography affirmed that FAK inhibitors induce cancer apoptosis [21,66,67,68]. PND-1186, a FAKi, inhibited 4T1 breast carcinoma tumor growth, which correlated with elevated apoptosis and caspase-3 activation [68]. A different FAKi, GSK2256098, suppresses FAK downstream signaling pathways, inducing apoptosis via caspase-9/PARP [69]. Our results confirmed that in BC cells, treatments with RA, FAKi (2 µM) and their combination induced an increase in the apoptosis markers active caspase-3 and cleaved PARP1.

When we assessed the expression of RARs, FAK and Paxillin by western blot, we observed that RA induces the downregulation of RARα, FAK and Paxillin. Coadministration with FAKi improves this downregulation. We have previously reported that RA treatment (72 h) promotes FAK downregulation [26]. Similarly, Dutta et al. (2009 and 2010) have demonstrated that RA treatment (20–30 μM, 24–48 h) on MDA-MB-231 and MCF-7 cells triggers the downregulation of FAK expression [70,71]. They also reported that the administration of RA increases RAR expression, but they did not differentiate between RAR isoforms [71].

We also study the phosphorylation/activation of FAK and its downstream protein Paxillin under our experimental conditions. We had previously reported that the administration of 1 µM RA for short periods (20 min) increased the activation/phosphorylation of FAK and Paxillin, inducing their nuclear relocalization in T-47D breast cancer cells [27]. In the current study, we demonstrated that prolonged treatment with RA, FAKi, and their combination inhibit FAK and Paxillin phosphorylation, preventing their activation. These findings also confirmed the inhibitory mechanism of FAKi and that the inhibition of FAK and Paxillin persist after a long treatment (72 h). 

One of the most intriguing actions of RA and FAKi is the control of the distribution and expression of FAK, overexpressed in the majority of breast cancer subtypes and critical for the invasion and metastasis process. In this work, we observed that RA and FAKi reduce FAK expression, achieving a higher diminishment when cells were exposed to the combined RA+FAKi treatment. Interestingly, we also provide evidence that RA, FAKi, and the combined treatment inhibit the relocalization of FAK to the plasmatic membrane, leaving cells with a static phenotype. This phenomenon was observed when the treatment of RA and RA+FAKi promoted nuclear FAK translocation. Our group and other colleagues have reported that the individual use of the RA and FAK inhibitor enables the FAK to shuttle between the cytoplasm and the nucleus [27,72,73,74]; however, the nuclear role of FAK is not completely clear. FAK in the cytoplasm controls proliferation, adhesion, migration, and invasion in a kinase-dependent manner, while FAK in the nucleus acts in a kinase-independent way to regulate gene expression [74]. We suggest that when FAK is not found at FAs, it is impossible to trigger the migratory and invasive processes. In agreement with our previous findings, we propose an interesting regulatory mechanism in cell adhesion and migration, which are affected by the subcellular localization of FAK [27]. 

Another important finding of this work is the correlation between in vitro and in vivo results. In a murine BC model, we found that the administration of RA plus FAKi reduces tumor volume from the tenth day after orthotopic inoculation. Furthermore, we demonstrated that mice administered with the combined treatment enhanced their survival compared to the control group. In accordance with this, other authors have demonstrated that RA inhibits the growth of LM3, MDA-MB-231, and MCF-7 orthotopic tumors [75,76,77]. Furthermore, we showed that the exposition of mice to the combined treatment decreases Ki-67 expression, indicating a more differentiated state and a reduction in tumor cellular proliferation. This result is consistent with previous works that demonstrated the molecular actions of RA and FAKi promoting tumor cell differentiation [78,79,80]. 

Finally, we evaluated the ability of RA and FAKi to inhibit tumor cell dissemination. RA administered as a single agent was effective in interrupting the spread of cancer cells to the lung. Previously, other groups have reported the ability of RA to inhibit metastasis [81,82,83]. In contrast, Berardi et al. (2014) and Bessone et al. (2020) did not observe differences between the control and RA-treated groups [75,84]; however, they placed the RA patch five days before inoculation while we placed it one week previous to the injection. One interesting point to highlight is that FAK is inhibited during the first few days, 7–10 days approximately, at the time of tumor formation, and it has a negative effect on tumor growth. We suggested that FAK inhibition reduces adherence and the effective colonization of tumor cells. Cells that fail to adhere colonize and adapt to the new environment, die and do not participate in tumor formation, and tumor volume is reduced. The same is valid for the experimental metastasis assay; FAK is inhibited during the first few days, at the time of cell colonization, and cells that fail to colonize and adapt to the new environment die and do not form metastatic nodules.

**Figure 6:**
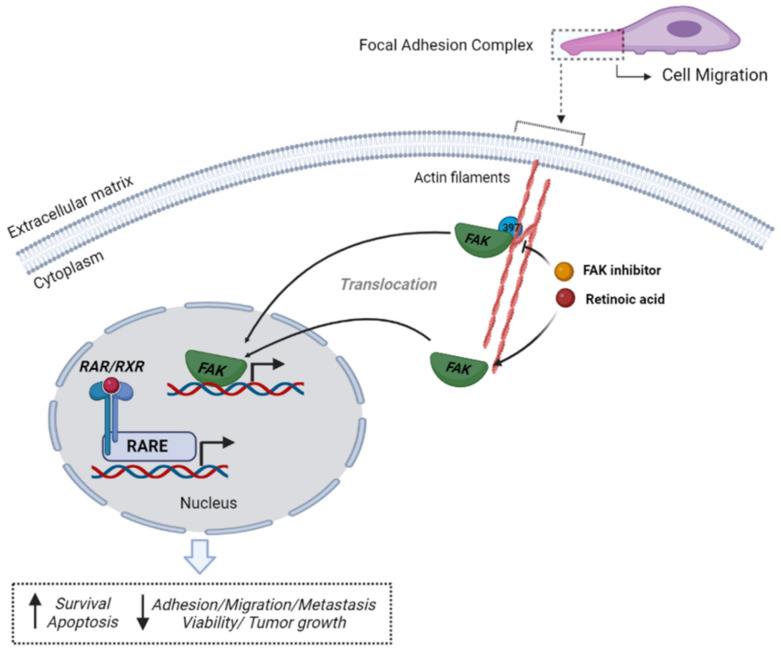
Schematic figure of molecular changes triggered by RA plus FAKi in BC. Retinoic acid (RA) and FAK inhibitor (FAKi) induces FAK translocation from focal adhesion sites and cytoplasm to the nucleus. In the nucleus, FAK acts as a transcription factor in a kinase-independent manner. In parallel, RA binds to receptors RXR/RARs regulating the transcription of target genes. As result, cell detachment occurs blocking cell migration and reducing the metastatic process. Likewise, cell viability diminished due to apoptosis induction decreasing tumor growth and increasing mice survival. Figure created by BioRender.

In conclusion, our study reveals that RA-resistance would reflect the deregulation of most of the RA-target genes involved in cell adhesion, migration, and invasion, including genes encoding components of the Src-FAK dependent pathway. Our study demonstrates that RA and FAKi are essential in disrupting BC tumor growth and metastatic dissemination in vitro and in vivo by controlling FAK expression, phosphorylation/activation, and localization. FAKi impaired critical phosphorylation of FAK in residue Tyr 397, which serves as a canonical docking site for other signaling kinases and scaffolding proteins. Consequently, due to the impediment of protein interactions, a dissociation in the activation of downstream oncogenic pathways occurs. The blockade of FAK phosphorylation induces FAK detachment from FAs and its translocation to the nucleus. FAK acts as a transcriptor factor in a kinase-independent way to regulate target gene expression (Figure 6). Additionally, we show that RA, FAKi and their combination induce the expression of critical facilitators of apoptosis such as active-caspase-3 and cleaved-PARP1. Despite the complexity of the retinoid signaling in BC, the approach based on RA administered in combination with FAKi may be an effective strategy for treating metastatic patients since we demonstrated that RA sensibility could be exacerbated with FAKi coadministration. The combined treatment would inhibit the malignant phenotype in BC cells by disrupting cancer cell spread. Our findings also support the concept that the multifunctional protein FAK is a promising target for cancer therapy. Clinical trials are currently investigating treatment strategies using FAK inhibitors combined with chemotherapy, targeted therapy, or immunotherapy to increase FAKi efficacy [23]. Thus, selecting patients who could benefit from FAKi treatment alone or combination strategies would be essential.

## Data Availability

The original contributions presented in the study are included in the article/supplementary material. Further inquiries can be directed to the corresponding author(s).

## Supplementary Materials

The following supporting information can be downloaded at: https://www.mdpi.com/article/10.3390/cells11192988/s1.
