# Supplementary figures and images for: Combination Treatment of Retinoic Acid Plus Focal Adhesion Kinase Inhibitor Prevents Tumor Growth and Breast Cancer Cell Metastasis
Source: Cells. 2022 Sep 26;11(19):2988. doi: 10.3390/cells11192988 (PMC9564078; doi:10.3390/cells11192988)

## Slide 1
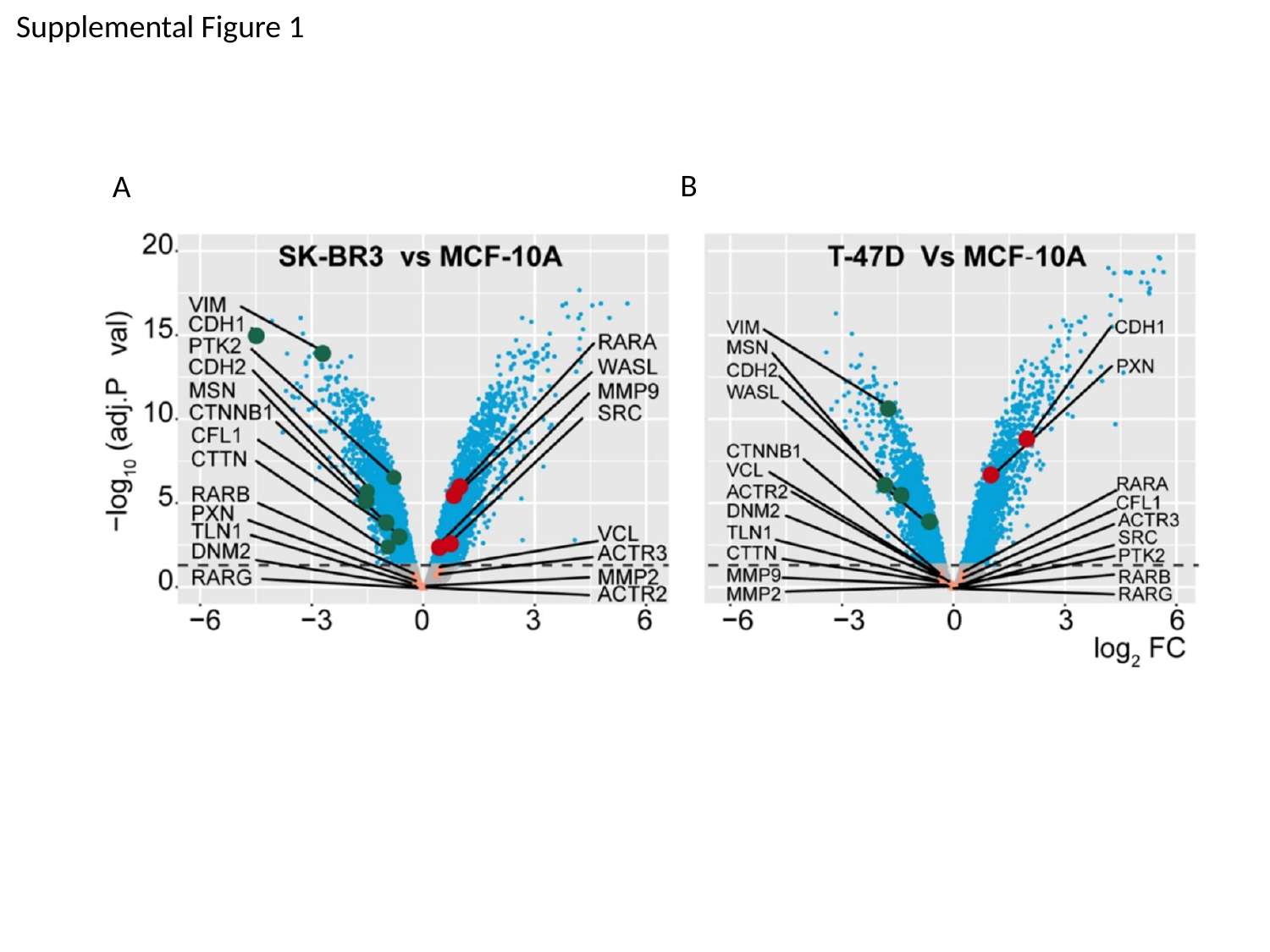

Supplemental Figure 1
B
A

## Slide 2
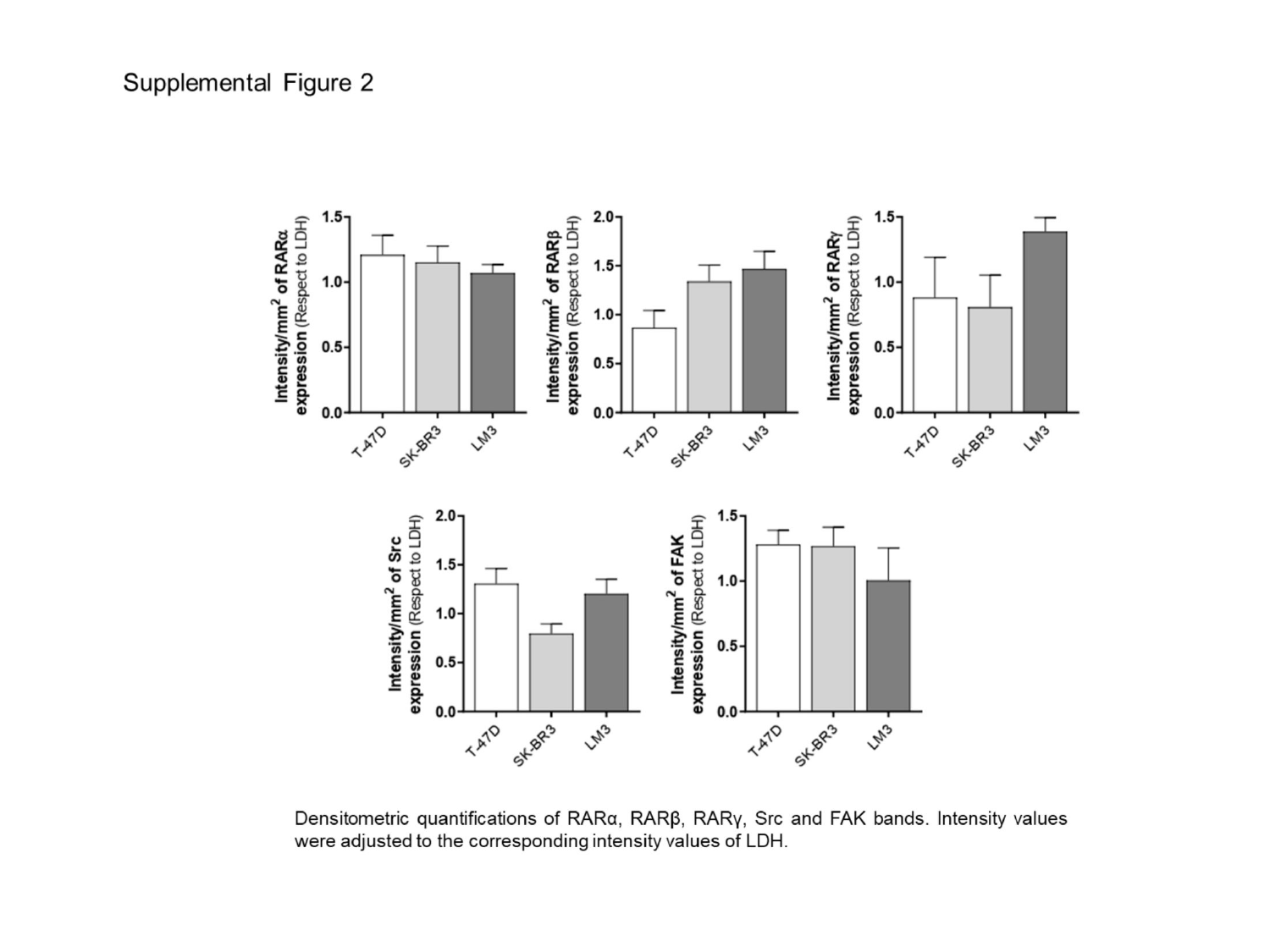

## Slide 3
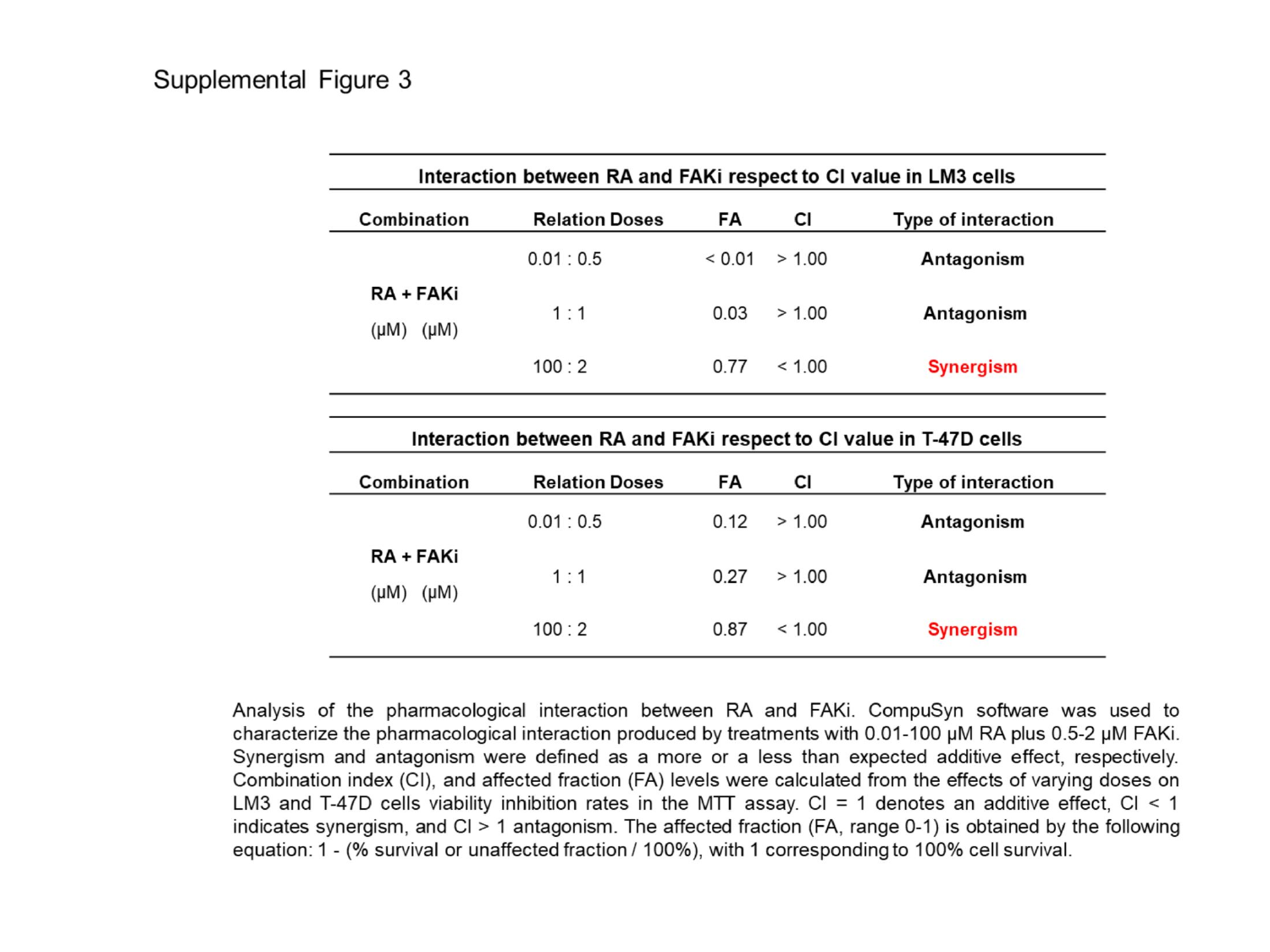

## Slide 4
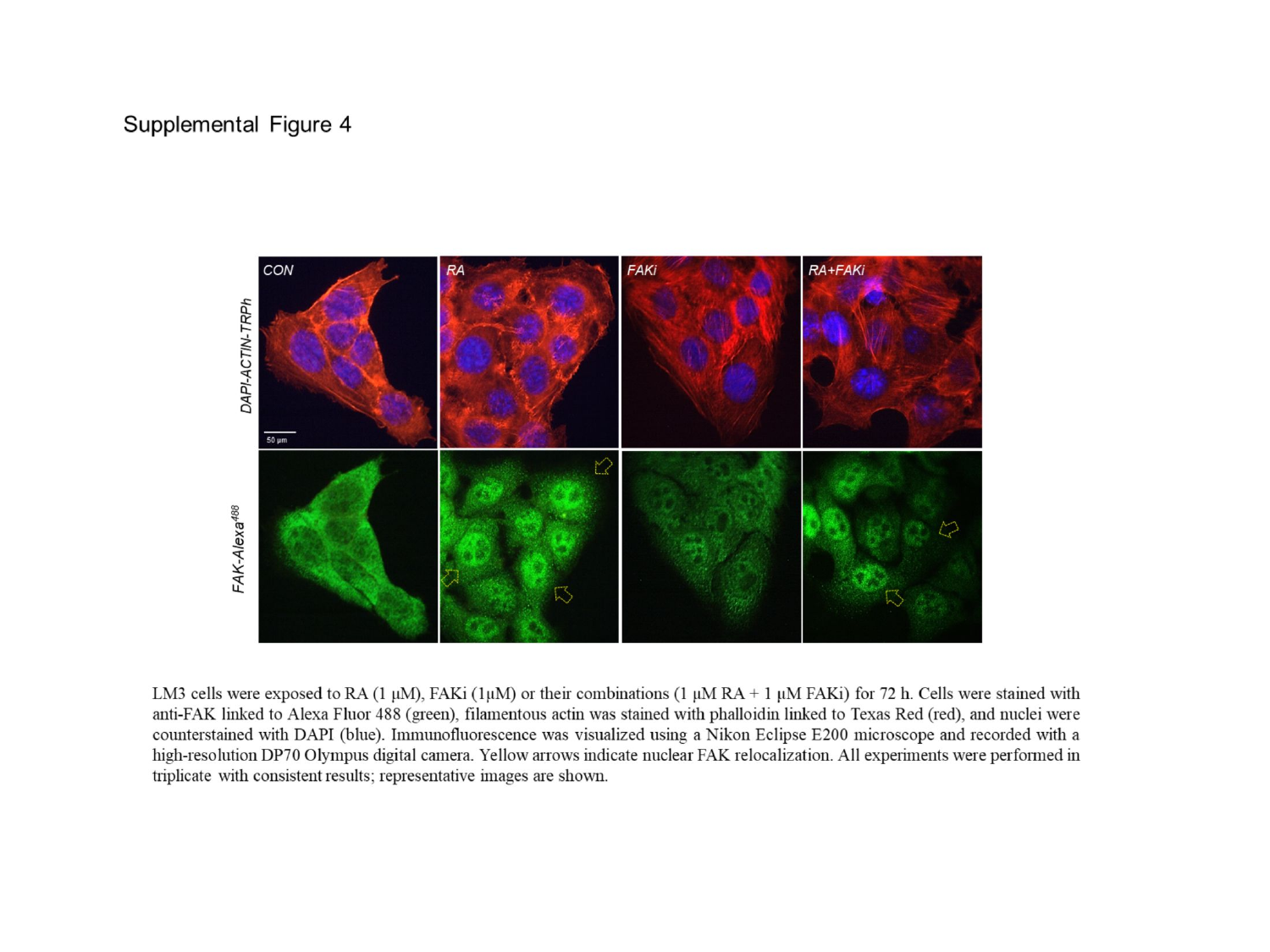

#

## Slide 5
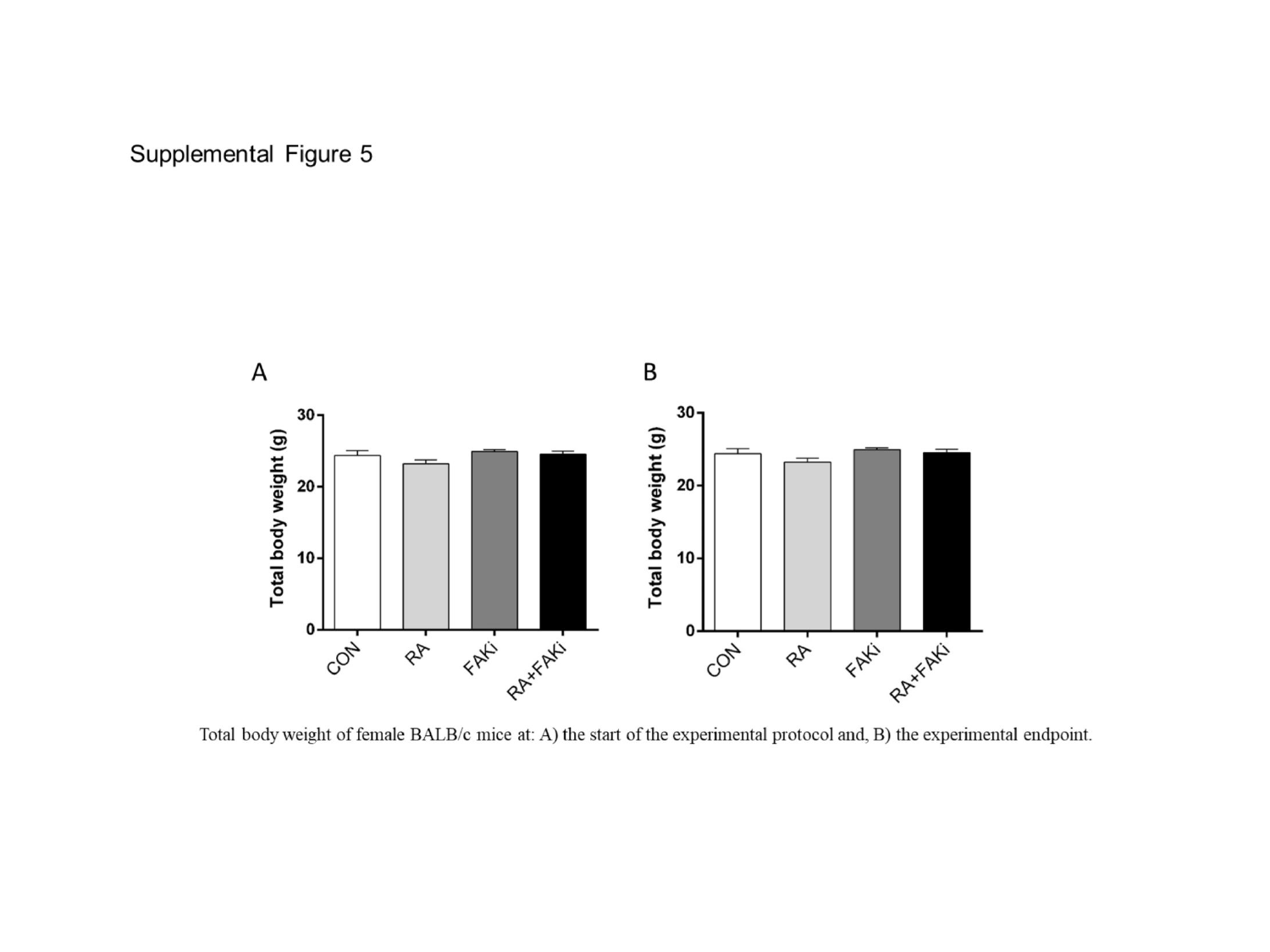

#

Supplement: Supplementary file 1 [file cells-11-02988-s001.zip › cells-1768909-supplementary.pptx]
